# Exploring dual diagnosis in opioid agonist treatment patients: a registry-linkage study in Czechia and Norway

**DOI:** 10.1186/s13722-024-00467-5

**Published:** 2024-05-14

**Authors:** Gabriela Rolová, Svetlana Skurtveit, Roman Gabrhelík, Viktor Mravčík, Ingvild Odsbu

**Affiliations:** 1 Department of Addictology, First Faculty of Medicine, Charles University, Apolinářská 4, Prague, 128 00 Czechia; 2 Department of Addictology, General University Hospital in Prague, Prague, Czechia; 3 Department of Chronic Diseases, Norwegian Institute of Public Health, Oslo, Norway; 4 Norwegian Centre for Addiction Research, University of Oslo, Oslo, Norway

**Keywords:** Opioid use disorder, Psychiatric comorbidity, Dual diagnosis, Registry-based study, Opioid agonist treatment

## Abstract

**Background:**

Knowledge of co-occurring mental disorders (termed ‘dual diagnosis’) among patients receiving opioid agonist treatment (OAT) is scarce. This study aimed (1) to estimate the prevalence and structure of dual diagnoses in two national cohorts of OAT patients and (2) to compare mental disorders between OAT patients and the general populations stratified on sex and standardized by age.

**Methods:**

A registry-linkage study of OAT patients from Czechia (*N* = 4,280) and Norway (*N* = 11,389) during 2010–2019 was conducted. Data on mental disorders (F00-F99; ICD-10) recorded in nationwide health registers were linked to the individuals registered in OAT. Dual diagnoses were defined as any mental disorder excluding substance use disorders (SUDs, F10-F19; ICD-10). Sex-specific age-standardized morbidity ratios (SMR) were calculated for 2019 to compare OAT patients and the general populations.

**Results:**

The prevalence of dual diagnosis was 57.3% for Czechia and 78.3% for Norway. In Czechia, anxiety (31.1%) and personality disorders (25.7%) were the most prevalent, whereas anxiety (33.8%) and depression (20.8%) were the most prevalent in Norway. Large country-specific variations were observed, e.g., in ADHD (0.5% in Czechia, 15.8% in Norway), implying differences in screening and diagnostic practices. The SMR estimates for any mental disorders were 3.1 (females) and 5.1 (males) in Czechia and 5.6 (females) and 8.2 (males) in Norway. OAT females had a significantly higher prevalence of co-occurring mental disorders, whereas SMRs were higher in OAT males. In addition to opioid use disorder (OUD), other substance use disorders (SUDs) were frequently recorded in both countries.

**Conclusions:**

Results indicate an excess of mental health problems in OAT patients compared to the general population of the same sex and age in both countries, requiring appropriate clinical attention. Country-specific differences may stem from variations in diagnostics and care, reporting to registers, OAT provision, or substance use patterns.

**Supplementary Information:**

The online version contains supplementary material available at 10.1186/s13722-024-00467-5.

## Introduction

Mental disorders are common among individuals with opioid use disorder (OUD) [1, 2]. The co-occurrence of mental disorders with OUD (also referred to as dual diagnosis) [3] has been associated with increased mortality, adverse physical health outcomes [4, 5], and poor psychosocial functioning [6]. Opioid agonist treatment (OAT) is considered a state-of-the-art intervention effective in reducing illicit substance use and improving the physical [7–9] as well as mental health [10–12] of patients with OUD. However, there is little research comprehensively assessing mental health among OAT patients [4, 13, 14].

Overall, approximately 40–90% of individuals with OUD are estimated to have co-occurring mental disorders [2, 4, 15–18], although large variations exist between countries as well as clinical settings [19]. In Europe, the epidemiological estimates are somewhat limited, but existing research shows that the prevalence of dual diagnosis in people with OUD is up to 40%. Depression (36%), anxiety (29%), attention-deficit/hyperactivity disorder (ADHD; 21%), post-traumatic stress disorder (PTSD; 18%), and antisocial personality disorders (34%) are among the most prevalent mental disorders [1]. Sex differences were reported, consistently showing higher rates of dual diagnosis in women compared to men [1, 2, 13, 20]. Other risk factors associated with a higher burden of mental disorders include frequent polydrug use [21], unstable living conditions, and socioeconomic vulnerability [13].

Individuals with dual diagnosis generally show higher psychopathological severity [22] and suicidality rates [23], more frequently engage in drug-related risky behavior [24], are more likely to receive treatment in hospital emergency departments [25] and experience greater psychosocial vulnerability (e.g., social exclusion, unemployment, homelessness, and incarceration) [26–29]. In terms of disability and premature deaths, co-occurring mental disorders represent a major predictor in people with SUDs [4, 5, 30], especially in younger age groups [31].

The research on co-occurring mental disorders in individuals receiving OAT is currently limited, particularly in Europe. Comparing across studies can be challenging, as they are inconsistent with regard to research settings, treatment outcomes, or assessment tools. Previous studies have predominantly focused on major mental disorders such as depression and anxiety [2, 16] while overlooking less prevalent disorders. Moreover, studies have revealed significant heterogeneity, with reported dual diagnosis in OAT patients ranging from 65 to 87% [4, 13, 14]. These studies are often based on selected and relatively small or non-European study populations, and the information on dual diagnoses largely relies on self-assessment tools. Consequently, these findings may not accurately represent the broader diagnostic and treatment practices.

In summary, there is a pressing need for new research to address the gaps in our existing knowledge, account for variations in nationwide prevalence rates, focus specifically on the unique OAT population in comparison to the general population and provide insights crucial for shaping future strategies in the prevention and treatment of co-occurring mental disorders among OAT patients.

The aim of the study was (1) to estimate the prevalence and structure of dual diagnoses in two national cohorts of OAT patients from Czechia and Norway, representing different treatment settings, and (2) to compare mental disorders between OAT patients and the general populations of Czechia and Norway standardized by age and sex.

## Methods

### Study design

This was a registry-linkage study on the national samples of OAT patients from Czechia and Norway in 2010–2019. The protocols for the overall comparative registry linkage studies [32, 33] and previous findings on somatic comorbidity and mortality in the same cohort of OAT patients can be found elsewhere [34, 35].

### Setting

The study was conducted in Czechia and Norway. The countries share similarities but also differences in terms of population characteristics, healthcare systems, and OAT provision (see also [34, 35]).

Both countries have publicly funded healthcare systems that ensure universal coverage for all citizens and legal residents. The countries have decentralized healthcare systems that comprise a combination of public and private healthcare providers. The financing of healthcare services largely relies on taxation and employer/employee contributions.

In terms of drug treatment services, both countries have well-established service networks for individuals with SUDs, including harm reduction programs and specialized outpatient and inpatient addiction treatment services. OAT is offered in Czechia and Norway with different levels of availability and affordability. In Czechia, methadone is available in specialized OAT clinics with varying levels of threshold and is fully covered by public funds. Office-based OAT with buprenorphine products is available, but only around 6% of primary care physicians provide this treatment [36]. In the study period, the majority of patients were required to pay the full price for the most buprenorphine prescriptions. In Norway, OAT is considered low-threshold and harm reduction-/public health-oriented, characterized by less restrictive eligibility and prescription criteria, with the costs entirely covered by public budgets.

### Study population and study period

The study population consisted of all individuals with at least one record of receiving OAT in Czechia and Norway between January 1, 2010–December 31, 2019. In total, 4,280 OAT patients from Czechia and 11,389 OAT patients from Norway were included in the study. The mean age of patients was calculated based on their age at the midpoint of the study period, i.e., in 2015.

When comparing with the general population, we excluded all OAT patients who died between 2010 and 2019 (Czechia: *N* = 267, Norway: *N* = 1,512). The age of the patients was calculated for 2019.

### Data sources and linkage

Multiple population-based health registers from Czechia and Norway were utilized. In each country, physicians are legally obliged to report patient data to these nationwide registers prospectively. The quality of the register data is considered high [37–39].

For Czechia, the National Register of Therapy of Drug Users (NRLUD) was used to identify OAT patients. The NRLUD collects demographic and treatment-related data of all individuals entering addiction treatment facilities in Czechia. Information on mental disorders (F00-F99, G47) was retrieved from the National Register of Hospitalized Patients (NRHOSP), which covers all completed hospitalizations, and the National Register of Reimbursed Health Services (NRHZS), covering outpatient mental healthcare.

For Norway, the Norwegian Prescription Database (NorPD) provided data on OAT medications dispensed and dispensation date [40]. Since Norway does not have a specific OAT registry, NorPD provided a proxy indication by identifying OAT patients based on filled prescriptions. This database identifies approximately 90% of OAT patients in Norway [41]. Opioids used for identifying OAT patients included methadone oral solution (Anatomical Therapeutic Chemical (ATC) code N07BC02) and high-dose buprenorphine tablets (≥ 2 mg sublingual tablets, N07BC01 (buprenorphine) or N07BC51 (buprenorphine-naloxone), all almost solely prescribed for the treatment of OUD in Norway. Mental disorder diagnoses were obtained from the Norwegian Patient Registry (NPR), which contains information on all patients receiving hospital-level care in both inpatient and outpatient facilities and acute and emergency services for mental and somatic illnesses.

In both countries, individual-level data from multiple registers were linked using the unique personal identification number (PIN) assigned to all residents in their respective countries. This enabled the linkage of patient records across various treatment episodes and registers. In order to de-identify the individuals, the PINs were replaced by project-specific IDs. Diagnoses are recorded according to the International Classification of Diseases, 10th revision (ICD-10).

### Outcomes

Dual diagnosis was defined as the presence of any mental health disorder other than SUDs (i.e., excluding F10-F19; ICD-10). Other mental disorders than SUDs were identified as F00-F09, F20-F99, and G47 (ICD-10). In the analysis, we included all diagnoses patients received during individual inpatient or specialist outpatient treatment episodes between January 1, 2010–December 31, 2019, regardless of the date of the first OAT entry. The reason for this is the chronic nature of OUD, high patient fluctuation in and out of OAT, as well as the inability to control OAT provision prior to 2010. To identify mental disorders, we selected only records that patients received in specialized and hospitalization care. Primary care records were excluded from the analysis to capture only specialist-confirmed diagnoses. In addition, we compared the mental disorders in OAT patients with sex-specific age-standardized estimates from the general population in 2019.

### Statistical analysis

The prevalence (%) of dual diagnoses was calculated as the number of OAT patients with at least one recorded dual diagnosis during the study period, divided by the total number of patients receiving OAT during the study period (2010–2019). The prevalence was calculated for the entire OAT population and stratified by sex. Other patient characteristics could not be utilized for stratification due to their unavailability in the data sets used in this study or optional recording in the registers, leading to incomplete reporting and high rates of missing values. Dual diagnoses were described overall (any mental disorder excl. SUD) and diagnostic groups of dual diagnoses according to ICD-10. Pearson’s chi-square test was used to test for differences in sex groups.

To compare the prevalence rates of selected mental disorders (Table 3) in OAT and general populations in Czechia and Norway, we calculated the sex-specific age-standardized morbidity ratios (SMR) and their 95% confidence intervals. This was done by dividing the observed number of prevalent morbidities (cases) in the study cohort by the expected number of cases. The general populations of Czechia and Norway were used as the reference populations. The expected numbers of cases were calculated using prevalence rates of investigated mental disorders in the general Czech and Norwegian population obtained from the outpatient and hospitalization registers (NRHZS and NRHOSP for Czechia and NPR for Norway) in 2019 by 10-year age groups. The analysis was performed for any mental disorders (excl. SUDs) and the three most frequent disorder categories. Since data available for the general populations were classified according to the ICD-10, to enable the SMR calculations, the main disorder categories were re-grouped to align with the ICD-10 diagnosis sections (Table 2). Statistical analyses were performed using R software and IBM SPSS Statistics 27.

## Results

### Baseline characteristics

In total, 15,669 patients who received OAT in Czechia (*N* = 4,280) and Norway (*N* = 11,389) between 2010 and 2019 were identified in the national registers (Table 1). One-third of the OAT populations in both countries were females. The mean age was higher for both females and males in the Norwegian cohort (Czechia: 35.8 years for males and 33.0 years for females; Norway: 42.8 years for males and 41.8 years for females). Methadone use was similar in both countries, but the Czech cohort had higher buprenorphine than buprenorphine-naloxone use compared to the Norwegian cohort. The majority of OAT patients in Czechia and Norway were diagnosed with OUD (F11) and/or polydrug use (F19) (Supplementary Table 1).

**Table 1:** Characteristics of the study population of opioid agonist treatment (OAT) patients in Czechia and Norway by sex during 2010–2019

	Czechia (*N* = 4,280)		Norway (*N* = 11,389)	
	Males	Females	Males	Females
Number of OAT patients (n, %)	2992 (69.9)	1288 (30.1)	8006 (70.3)	3383 (29.7)
**Age at 2015**				
Mean (SD)	35.8 (6.6)	33.0 (6.5)	43.8 (10.2)	42.8 (10.3)
**First OAT medication**				
Methadone (n, %)	946 (31.8)	446 (34.6)	2723 (34.0)	1215 (35.9)
Buprenorphine (n, %)	1225 (40.9)	512 (39.8)	2557 (31.9)	1142 (33.8)
Buprenorphine-naloxone (n, %)	821 (27.4)	330 (25.6)	2726 (34.0)	1026 (30.3)

SD = standard deviation

### Dual diagnosis in OAT patients

In Czechia, 57.3% of OAT patients had the dual diagnosis (Table 2, Supplementary Table 2). In Norway, the corresponding proportion was 78.3%. The most common disorder category was phobia and other anxiety disorders in both countries (Czechia: 31.1%, Norway: 33.8%), followed by disorders of adult personality and behavior (25.7%) in Czechia and depressive and related mood disorders (20.8%) in Norway. Norway had a higher prevalence of co-occurring mental disorders across most disorder categories. The largest differences were found for disorders of adult personality and behavior and hyperkinetic disorders; 14.0% of OAT patients in Czechia and 1.4% of OAT patients in Norway were diagnosed with somatoform and other disorders, whereas 0.8% and 15.8%, respectively, were diagnosed with hyperkinetic disorders.

**Table 2:** Prevalence of co-occurring mental disorders among opioid agonist treatment (OAT) patients in Czechia and Norway in total and stratified by sex

		Czechia							Norway						
		Total (*N* = 4,280)		Males (*n* = 2,992)		Females (*n* = 1,288)			Total (*N* = 11,389)		Males (*n* = 8,006)		Females (*n* = 3,383)		
Description	ICD-10	*n*	%	*n*	%	*n*	%	*p*	*n*	%	*n*	%	*n*	%	*p*
Dual diagnosis (excl. SUDs)*	F00-F99 (excl. F10-F19)	2454	57.3	1680	56.1	774	60.1	0.017	8915	78.3	6200	77.4	2715	80.3	0.001
Organic, including symptomatic, mental disorders	F00-F09	171	4.0	130	4.3	41	3.2	0.088	516	4.5	384	4.8	132	3.9	0.036
Schizophrenia and related disorders	F20-F29	247	5.8	181	6.0	66	5.1	0.253	1210	10.6	868	10.8	342	10.1	0.246
Bipolar disorders	F30-F31	91	2.1	48	1.6	43	3.3	< 0.001	436	3.8	256	3.2	180	5.3	< 0.001
Depressive and related mood disorders	F32-F34, F38-F39	513	12.0	331	11.1	182	14.1	0.005	2371	20.8	1586	19.8	785	23.2	< 0.001
Phobia and other anxiety disorders	F40-F43	1332	31.1	858	28.7	474	36.8	< 0.001	3850	33.8	2484	31.0	1366	40.4	< 0.001
Somatoform and other disorders	F45, F48	327	7.6	216	7.2	111	8.6	0.117	162	1.4	84	1.0	78	2.3	< 0.001
Eating disorders	F50	24	0.6	3	0.1	21	1.6	< 0.001	145	1.3	22	0.3	123	3.6	< 0.001
Sleep disorders	F51, G47	177	4.1	127	4.2	50	3.9	0.616	633	5.6	460	5.7	173	5.1	0.179
Sexual dysfunction	F52	40	0.9	35	1.2	5	0.4	0.014	26	0.2	18	0.2	8	0.2	0.905
Disorders of adult personality and behaviour	F60-F69	1101	25.7	790	26.4	311	24.1	0.127	1683	14.8	1064	13.3	619	18.3	< 0.001
Hyperkinetic disorders	F90	22	0.5	16	0.5	6	0.5	0.822	1797	15.8	1294	16.2	503	14.9	0.083
Unspecified mental disorder	F99	363	8.5	259	8.7	104	8.1		1235	10.8	853	10.7	382	11.3	

SUD = substance use disorder

p = p-value for differences between males and females

*Any mental disorder (excl. SUDs)

### Sex differences

In Czechia, 60.1% of females had dual diagnosis compared to 56.1% of males (Table 2, Supplementary Table 3). For Norway, the corresponding proportions were 80.3% of females and 77.4% of males. In both countries, females had significantly higher prevalence rates of most mental disorder categories, except for sexual dysfunction in Czechia (1.2% of males vs. 0.4% of females) and organic mental disorders in Norway (4.8% of males vs. 3.9% of females), which were more common in men. The largest sex differences were in phobia and other anxiety disorders, which were more common in females compared to males in Czechia (28.7% of males vs. 36.8% of females) as well as in Norway (31.0% of males vs. 40.4% of females).

Norway generally had higher prevalence rates across most mental disorder categories than Czechia. The largest differences were in hyperkinetic disorders and depressive and related mood disorders for both sex groups. In Norway, 16.2% of males and 14.9% of females were diagnosed with hyperkinetic disorders, while it was 0.5% in both males and females in Czechia. For depressive and related mood disorders, 19.8% of males and 23.2% of females in Norway were diagnosed, while it was 11.1% and 14.1%, respectively, in Czechia. In addition, a notably higher proportion of males in Czechia had disorders of adult personality and behavior than in Norway (26.4% vs. 13.3%).

### One-year sex-specific age-standardized morbidity ratios

In Czechia, the SMR for all mental disorders other than SUDs was 5.1 for males and 3.1 for females (Table 3). In Norway, the corresponding estimates were 8.2 for males and 5.6 for females. The highest SMRs were found for disorders of adult personality and behavior; the SMRs were 23.9 for males and 16.8 for females in Czechia and 9.2 for males and 10.1 for females in Norway.

**Table 3:** One-year sex-specific age-standardized morbidity ratios (SMRs) with 95% confidence intervals (CIs) of selected mental disorders for opioid agonist treatment (OAT) patients in Czechia and Norway in 2019

		Czechia								Norway							
		Males (*n* = 2,776)				Females (*n* = 1,237)				Males (*n* = 6,846)				Females (*n* = 3,031)			
Description	ICD-10	Observed *n*	Expected *n*	Age-standardized SMR	95% CI	Observed *n*	Expected *n*	Age-standardized SMR	95% CI	Observed *n*	Expected *n*	Age-standardized SMR	95% CI	Observed *n*	Expected *n*	Age-standardized SMR	95% CI
Any mental disorder (excl. SUD)	F00-F99 (excl. F10-F19)	585	115.6	5.1	(4.7, 5.5)	242	77	3.1	(2.7, 3.5)	2263	276.9	8.2	(7.8, 8.5)	1052	186.2	5.6	(5.3, 6)
Mood (affective) disorders	F30-F39	77	17.8	4.3	(3.4, 5.3)	42	14.3	2.9	(2.1, 3.8)	291	96.3	3	(2.7, 3.4)	161	70.3	2.3	(1.9, 2.6)
Neurotic, stress-related and somatoform disorders	F40-F48	273	51.0	5.4	(4.7, 6.0)	137	45.4	3	(2.5, 3.5)	646	110.7	5.8	(5.4, 6.3)	408	102.4	4.0	(3.6, 4.4)
Disorders of adult personality and behaviour	F60-F69	231	9.7	23.9	(20.8, 27.0)	67	4	16.8	(12.8, 20.8)	286	31.1	9.2	(8.1, 10.3)	191	19	10.1	(8.6, 11.5)

SUD = substance use disorder

SMR = standardized morbidity ratio

## Discussion

This study found a high prevalence of dual diagnosis in OAT patients with country-specific differences in the main categories of mental disorders. In Czechia, personality disorders and anxiety were the most common diagnostic categories, while depression and anxiety were the most common in Norway. OAT females had a significantly higher prevalence of co-occurring mental disorder diagnoses, whereas SMRs were higher in males.

The overall prevalence of dual diagnosis was 57.3% for Czechia and 78.3% for Norway. Previous studies have found co-occurring mental disorders in 65–87% of patients in OAT [4, 13, 14]. The heterogeneity in prevalence estimates across existing studies is presumed to be partially attributed to methodological differences [1, 42] and socio-cultural aspects [28]. Nevertheless, the consistent observation of prevalence rates well exceeding 50% elsewhere across studies indicates a considerable burden of co-occurring mental disorders in this population. In addition, in our previous study [34], we found a relatively high prevalence of somatic morbidity in the same population of OAT patients, indicating an overall high complex health burden in this population.

The observed differences in rates of diagnosed mental disorders may be influenced by varying screening, diagnosis, and healthcare practices in the two countries. First of all, while sharing similar healthcare systems with insurance coverage, Czechia and Norway are characterized by unique mental health cultural attitudes and service development, potentially impacting the screening and diagnostics of mental disorders. Norway has one of the highest proportion of mental healthcare profesionals per capita globally, well-developed mental health research and medical education, and regularly implements updated treatment services [43, 44]. Conversely, Czechia’s spending on mental health falls below the European Union average, along with a higher burden of mental health-related stigma, largely centralized and inadequate mental health service system, and a lack of mental health research [45–47]. These disparities could lead to less attention on assessing and diagnosing comorbid mental disorders in the Czech OAT cohort. Furthermore, since primary health care records were excluded in both countries, this may affect observed prevalence rates of those dual diagnoses likely treated in the general outpatient setting, as well as country differences in the provision and accessibility of general and specialized care. In Czechia, it may be that general practitioners are more likely to screen and diagnose patients for mental disorders rather than refer them to specialized mental healthcare. Conversely, in Norway, there may be a tendency for patients to be diagnosed with mental disorders more often in specialized healthcare settings. There is also a possibility that OAT patients in Norway have better access to mental healthcare, resulting in a higher frequency of diagnosed mental disorders.

On the contrary, Norwegian more inclusive approach to OAT may have contributed to the higher reported prevalence of mental disorders among OAT patients in Norway. The Norwegian low-threshold approach has proven effective, contributing to the wide availability of OAT to ensure that as many people with OUD as possible have access to treatment [48]. In Czechia, low capacity, limited affordability, high selectivity, and the adoption of strict eligibility and prescription criteria might have resulted in more stable individuals with less severe comorbidities entering OAT [49]. The 1.5–2 times higher prevalence of schizophrenia and related disorders, bipolar disorders, and depressive and related disorders in Norway further indicates that individuals with severe mental disorders might be excluded from OAT in Czechia.

Interestingly, we found a large difference in hyperkinetic disorders between the two countries. While ADHD was common in Norwegian OAT patients (15.8%), it was diagnosed much less frequently in Czech OAT patients (0.5%). This is in sharp contrast to previous research that found ADHD symptoms in 51% of individuals in treatment for SUDs in Czechia using a self-report screening tool [50]. Similarly, the proportion of hyperkinetic disorders in Norway was lower compared to previous studies reporting ADHD symptoms in 33% [51] and 45% [52] of OAT patients screened for ADHD. A study from the Netherlands found ADHD in 35.2% of long-term OAT patients [53]. These findings could indicate that hyperkinetic disorders have been largely unrecognized in Czech OAT patients and likely underdiagnosed in Norway as well. Unspecific symptoms, misdiagnosis for co-occurring mental disorders with similar manifestations, and low awareness among physicians can mask or underestimate the prevalence of ADHD diagnoses in these patients [52, 54].

Moreover, Norway showed substantially higher rates of diagnosed concurrent cannabis, sedatives/hypnotics, cocaine, and stimulant use compared to Czechia. Rather than indicating more frequent polydrug use or severe substance use issues in Norway, this might result from underreporting in Czech registers or strict prescription and eligibility criteria for OAT in Czechia. These may involve discontinuation if there is illicit drug use revealed [49], so patients hide their concurrent drug use to avoid strict oversight and the threat of discontinuation.

The one-year prevalence of dual diagnosis among OAT patients was 19.6% for Czechia and 32.9% for Norway (data not shown). The SMR estimates were 3.1 (females) to 5.1 (males) times higher for Czech OAT patients and 5.6 (females) to 8.2 (males) times higher for Norwegian OAT patients compared to the general non-OAT populations of corresponding sex and age groups. We found a high prevalence of anxiety, depression, and personality disorders among OAT patients in both countries. This is consistent with previous research showing that these mental disorders are among the most frequently co-occurring in individuals with OUDs [1, 18]. The sub-analysis provided further evidence that OAT patients are disproportionately affected by these mental disorders, particularly personality disorders, compared to the general population.

Treating OUD in individuals with severe personality disorders could be challenging for often more severe drug use profiles and unfavorable treatment outcomes [55]. The literature is consistent that personality disorders, particularly borderline (BPD) and antisocial disorders (ASPD), frequently co-occur with OUD [1]. The intersection between these disorders and opioid use is emotional dysregulation and impulsivity, and potentially shared common neurobiological substrate as dysregulation of the endogenous opioid system (EOS) and µ-opioid receptors has been given a central role in the psychopathology of both BPD and ASPD. The key role of EOS in brain reward circuits, together with opioid-stimulating potential, likely contributes to the increased vulnerability of individuals with BPD and ASPD to opioid use and dependence [56, 57]. Consequently, consistent with the self-medication theory [58], µ-opioid receptor agonists used for OAT may potentially alleviate the symptoms of personality disorders. In addition, psychotherapeutic approaches such as dialectical behavior therapy (DBT) or dynamic deconstructive psychotherapy (DDP) have been commonly recommended for personality disorders. Still, robust evidence of their efficacy in OAT patients is lacking [55].

The higher prevalence of dual diagnosis in females with OUD is well-established in the scientific literature [1, 2, 13, 20]. The evidence suggests the greater vulnerability of females to mental health issues, possibly due to a combination of biological, psychosocial, and environmental factors [60, 60]. Our results showed that a significantly higher proportion of females than males in both countries were affected by co-occurring mental disorders overall and across most main disease categories. However, compared to the general populations, OAT females had generally lower SMR estimates than men. This could reflect a higher prevalence of mental disorders in females in general [61, 62]. Therefore, relative to the general population, the excess risk of mental disorders was more pronounced for males than females in OAT.

The risk differences further highlight the importance of adopting a sex-sensitive approach to address the specific needs of both males and females in OAT. This may involve considering flexible treatment regimes, medication dosing, complementary health and social services, and men/women-only programs [63–65]. Women are particularly likely to face barriers to seeking SUD treatment due to increased experiences with stigma, socio-economic vulnerability, sexual and physical violence, and potential caregiving responsibilities [29, 66]. The extent to which OAT services respond to the specific needs of males and females in terms of interventions provided is currently not known in either Czechia or Norway. Sex-specific interventions in other European countries lack evidence of effectiveness, creating further research opportunities [65].

Our findings warrant further research and clinical attention to the mental health of patients receiving OAT. The demonstrated clinical benefits of OAT in improving mental health outcomes independently of adjunctive psychosocial intervention [10] highlight the need to improve access to treatment for as many as possible. Setting a high threshold for OAT may disadvantage individuals with decompensated dual diagnoses from receiving appropriate treatment or exclude them altogether [48]. In addition, the superiority of OAT over placebo/waitlist and abstinence-based approaches regarding mental health outcomes underscores the benefits of long-term treatment retention and adherence [10]. While the evidence on the causal relationship between dual diagnosis and patient drop-out from OAT has not been conclusive [2, 67], there are clear indications that the presence of dual diagnosis significantly increases the odds of OAT termination by an addiction facility [68]. The consequences may be far-reaching, leading to subsequent re-deterioration of health outcomes and premature death [4, 5, 30, 69].

The high prevalence and variability of dual diagnosis among OAT patients underline the critical need for prioritizing psychiatric assessment and patient-centred treatment of co-occurring mental disorders to reduce the associated risks [4–6]. The evidence suggests that integrated care models involving both SUD and psychiatric treatment are clinically more effective than standard non-integrated approaches to managing dual diagnoses. The key advantage of integrating pharmacological, psychotherapeutic, and social interventions within a single service appears to be the reduction of barriers caused by care fragmentation [29, 70]. However, some literature suggests that the evidence supporting the efficacy of integrated treatment is limited, particularly concerning European OAT modalities [26].

### Strengths and limitations

This research introduces a standardized approach using the ICD-10 classification system to study dual diagnosis in nationwide cohorts of OAT patients, enabling more reliable and meaningful comparisons of results. A major strength of the study is the use of prospectively collected nationwide register data of high quality linked on the individual level, which provides us the opportunity to study national, unselected populations of OAT patients in both countries. We could also explore sex differences and have an insight into mental ill-health in females, who were often underrepresented in earlier studies of OAT patients. Another strength is utilizing data on mental disorders for SMR calculations from the same registers for the study cohorts and the general populations, minimizing potential biases from using different data sources.

This study had some limitations that need to be considered. First, we did not control the temporality; therefore, we could not explore the causality between OUD and mental disorders. Second, the prevalence of mental disorders was calculated for the entire study period, regardless of the date of OAT initiation, which might lead to misclassification bias. On the other hand, this is justifiable given the largely chronic nature of both SUD and mental health disorders. Third, in both countries, we excluded primary care data to capture more severe cases diagnosed in specialist healthcare and to reduce the risk of misdiagnosis. This could have led to some milder conditions being omitted in our analysis; therefore, the prevalence found in our study may be underestimated. Fourth, some observed differences in the prevalence of co-occurring mental disorders could be partially attributed to different clinical characteristics of patients, as we could not adjust for the duration of opioid use, the severity of OUD, and time spent in OAT.

Some differences may be due to varying coding practices and data sources in the two countries. While the patient data recorded in the registers are similar, each country creates, maintains, and administers its registers differently. Merging these registry datasets was not feasible for data protection and legal reasons. Since Norway lacks a dedicated OAT registry, the Norwegian cohort was identified using prescriptions by proxy indication from NorPD. It is estimated that 90% of patients dispensed OAT medication were registered in the NorPD [41]. The Czech cohort was directly selected from the OAT registry – the NRLUD. In addition, underreporting of office-based buprenorphine treatment in the NRLUD persists in Czechia despite legal requirements. This has an important implication – while in Norway, all patients on OAT medicines are sampled regardless of the special or general character of the treatment unit, in Czechia, patients from rather specialized centers were sampled, potentially leading to the omission of some office-based buprenorphine patients from the analysis [35]. Nevertheless, the potential loss of OAT cases is estimated as not compromising the nationwide coverage of the registers and dual diagnosis prevalence estimates.

The Czech NRLUD register for OAT has not been validated for clinical coverage, potentially limiting considerations on the accuracy and completeness of patient records. The validity of other Czech and Norwegian registers has been reported to be high [37–40].

## Conclusions

OAT patients suffer from a higher burden of dual diagnoses that require further attention from clinicians and researchers. Country-specific differences in diagnosed mental disorders could prompt treatment services to revise their current approaches, which may help to tailor the treatment according to patients’ needs. The findings emphasize the need for routine psychiatric assessment, tailored patient-centered care addressing specific psychosocial needs, and a sex-sensitive approach among OAT patients.

### Electronic supplementary material

Supplementary Material 1

## Data Availability

The register-based cohorts are based on individual-level data from national health registers. The authors are not allowed, by law, to publicly share this data. Therefore, the authors cannot make this data fully available to the public. The authors may share statistical code upon request.
