# Supplementary material for: Exploring dual diagnosis in opioid agonist treatment patients: a registry-linkage study in Czechia and Norway
Source: Addict Sci Clin Pract. 2024 May 14;19:37. doi: 10.1186/s13722-024-00467-5 (PMC11092244; doi:10.1186/s13722-024-00467-5)
Supplement: Supplementary file 1 — Supplementary Material 1 [file 13722_2024_467_MOESM1_ESM.docx]

**Supplementary Table 1** Prevalence of substance use disorder diagnoses (ICD-10 codes F10-F19) among opioid agonist treatment (OAT) patients in Czechia and Norway (2010–2019)

|  |  | **Czechia (N = 4,280)** | | **Norway (N = 11,389)** | |
| --- | --- | --- | --- | --- | --- |
| **Diagnosis description** | **ICD-10** | **n** | **%** | **n** | **%** |
| Alcohol and substance abuse disorders excl. tobacco | F10-F19 (excl. F17) | 4081 | 95.4 | 11372 | 99.9 |
| Use of alcohol | F10 | 1039 | 24.3 | 2849 | 25.0 |
| Use of opioids | F11 | 3940 | 92.1 | 11215 | 98.5 |
| Use of cannabinoids | F12 | 262 | 6.1 | 4315 | 37.9 |
| Use of sedatives or hypnotics | F13 | 414 | 9.7 | 6353 | 55.8 |
| Use of cocaine | F14 | 46 | 1.1 | 316 | 2.8 |
| Use of other stimulants, including caffeine | F15 | 889 | 20.8 | 4346 | 38.2 |
| Use of hallucinogens | F16 | 129 | 3.0 | 247 | 2.2 |
| Use of volatile solvents | F18 | 56 | 1.3 | 97 | 0.9 |
| Multiple drug use and use of other psychoactive substances | F19 | 2719 | 63.5 | 6922 | 60.8 |

**Supplementary Table 2** Prevalence of co-occurring mental disorders among opioid agonist treatment (OAT) patients in Czechia and Norway (2010–2019)

|  |  | **Czechia (N = 4,280)** | | **Norway (N = 11,389)** | |
| --- | --- | --- | --- | --- | --- |
| **Description*** | **ICD-10** | **n** | **%** | **n** | **%** |
| Dual diagnosis* | F00-F99 (excl. F10-F19) | 2454 | 57.3 | 8915 | 78.3 |
| Organic, including symptomatic, mental disorders | F00-F09 | 171 | 4.0 | 516 | 4.5 |
| Schizophrenia and related disorders | F20-F29 | 247 | 5.8 | 1210 | 10.6 |
| Bipolar disorders | F30-F31 | 91 | 2.1 | 436 | 3.8 |
| Depressive and related mood disorders | F32-F34, F38-F39 | 513 | 12.0 | 2371 | 20.8 |
| Depressive episode | F32 | 412 | 9.6 | 1499 | 13.2 |
| Recurrent depressive disorder | F33 | 121 | 2.8 | 1114 | 9.8 |
| Persistent mood [affective] disorders | F34 | 43 | 1.1 | 195 | 1.7 |
| Other mood [affective] disorders | F38 | 0 | 0.0 | 12 | 0.1 |
| Unspecified mood [affective] disorders | F39 | 5 | 0.1 | 50 | 0.4 |
| Phobia and other anxiety disorders | F40-F43 | 1332 | 31.1 | 3850 | 33.8 |
| Phobic anxiety disorders | F40 | 112 | 2.6 | 1227 | 10.8 |
| Other anxiety disorders | F41 | 636 | 14.9 | 1786 | 15.7 |
| Obsessive-compulsive disorder | F42 | 37 | 0.9 | 179 | 1.6 |
| Reaction to severe stress and adjustment disorders | F43 | 912 | 21.3 | 2008 | 17.6 |
| Somatoform and other disorders | F45, F48 | 327 | 7.6 | 162 | 1.4 |
| Eating disorders | F50 | 24 | 0.6 | 145 | 1.3 |
| Sleep disorders | F51, G47 | 177 | 4.1 | 633 | 5.6 |
| Sexual dysfunction | F52 | 40 | 0.9 | 26 | 0.2 |
| Disorders of adult personality and behaviour | F60-F69 | 1101 | 25.7 | 1683 | 14.8 |
| Specific personality disorders | F60 | 685 | 16.0 | 1339 | 11.8 |
| Mixed and other personality disorders | F61 | 588 | 13.7 | 248 | 2.2 |
| Enduring personality changes, not attributable to brain damage and disease | F62 | 12 | 0.3 | 180 | 1.6 |
| Habit and impulse disorders | F63 | 46 | 1.1 | 57 | 0.5 |
| Gender identity disorders | F64 | 2 | 0.0 | 6 | 0.1 |
| Disorders of sexual preference | F65 | 0 | 0.0 | 2 | 0.0 |
| Psychological and behavioural disorders associated with sexual development and orientation | F66 | 5 | 0.1 | 4 | 0.0 |
| Other disorders of adult personality and behaviour | F68 | 8 | 0.2 | 19 | 0.2 |
| Unspecified disorder of adult personality and behaviour | F69 | 0 | 0.0 | 18 | 0.2 |
| Hyperkinetic disorders | F90 | 22 | 0.5 | 1797 | 15.8 |
| Unspecified mental disorder | F99 | 363 | 8.5 | 1235 | 10.8 |

SUD = substance use disorder

*Any mental disorder (excl. SUDs)

**Supplementary Table 3** Prevalence of co-occurring mental disorders among opioid agonist treatment (OAT) patients in Czechia and Norway stratified by sex (2010–2019)

|  |  | **Czechia (N=4,280 )** | | | | **Norway (N= 11,389)** | | | | |
| --- | --- | --- | --- | --- | --- | --- | --- | --- | --- | --- |
|  |  | **Males (n = 2,992 )** | | **Females (n = 1,288 )** | | | **Males (n = 8,006)** | | **Females (n = 3,383)** | |
| **Description** | **ICD-10** | **n** | **%** | **n** | **%** | | **n** | **%** | **n** | **%** |
| Dual diagnosis* | F00-F99 (excl. F10-F19) | 1680 | 56.1 | 774 | 60.1 | | 6200 | 77.4 | 2715 | 80.3 |
| Organic, including symptomatic, mental disorders | F00-F09 | 130 | 4.3 | 41 | 3.2 | | 384 | 4.8 | 132 | 3.9 |
| Schizophrenia and related disorders | F20-F29 | 181 | 6.0 | 66 | 5.1 | | 868 | 10.8 | 342 | 10.1 |
| Bipolar disorders | F30-F31 | 48 | 1.6 | 43 | 3.3 | | 256 | 3.2 | 180 | 5.3 |
| Depressive and related mood disorders | F32-F34, F38-F39 | 331 | 11.1 | 182 | 14.1 | | 1586 | 19.8 | 785 | 23.2 |
| Depressive episode | F32 | 266 | 8.9 | 146 | 11.3 | | 1018 | 12.7 | 481 | 14.2 |
| Recurrent depressive disorder | F33 | 72 | 2.4 | 49 | 3.8 | | 721 | 9.0 | 393 | 11.6 |
| Persistent mood [affective] disorders | F34 | 30 | 1.0 | 15 | 1.2 | | 143 | 1.8 | 52 | 1.5 |
| Other mood [affective] disorders | F38 | 0 | 0.0 | 0 | 0.0 | | 9 | 0.1 | 3 | 0.1 |
| Unspecified mood [affective] disorders | F39 | 4 | 0.1 | 1 | 0.1 | | 26 | 0.3 | 24 | 0.7 |
| Phobia and other anxiety disorders | F40-43 | 858 | 28.7 | 474 | 36.8 | | 2484 | 31.0 | 1366 | 40.4 |
| Phobic anxiety disorders | F40 | 75 | 2.5 | 37 | 2.9 | | 834 | 10.4 | 393 | 11.6 |
| Other anxiety disorders | F41 | 398 | 13.3 | 238 | 18.5 | | 1184 | 14.8 | 602 | 17.8 |
| Obsessive-compulsive disorder | F42 | 20 | 0.7 | 17 | 1.3 | | 104 | 1.3 | 75 | 2.2 |
| Reaction to severe stress and adjustment disorders | F43 | 578 | 19.3 | 334 | 25.9 | | 1187 | 14.8 | 821 | 24.3 |
| Somatoform and other disorders (e.g. neurasthenia) | F45, F48 | 216 | 7.2 | 111 | 8.6 | | 84 | 1.0 | 78 | 2.3 |
| Eating disorders | F50 | 3 | 0.1 | 21 | 1.6 | | 22 | 0.3 | 123 | 3.6 |
| Sleep disorders | F51, G47 | 127 | 4.2 | 50 | 3.9 | | 460 | 5.7 | 173 | 5.1 |
| Sexual dysfunction | F52 | 35 | 1.2 | 5 | 0.4 | | 18 | 0.2 | 8 | 0.2 |
| Disorders of adult personality and behaviour | F60-F69 | 790 | 26.4 | 311 | 24.1 | | 1064 | 13.3 | 619 | 18.3 |
| Specific personality disorders | F60 | 448 | 15.0 | 237 | 18.4 | | 818 | 10.2 | 521 | 15.4 |
| Mixed and other personality disorders | F61 | 468 | 15.6 | 120 | 9.3 | | 174 | 2.2 | 74 | 2.2 |
| Enduring personality changes, not attributable to brain damage and disease | F62 | 9 | 0.3 | 3 | 0.2 | | 114 | 1.4 | 66 | 2.0 |
| Habit and impulse disorders | F63 | 34 | 1.1 | 12 | 0.9 | | 41 | 0.5 | 16 | 0.5 |
| Gender identity disorders | F64 | 2 | 0.1 | 0 | 0.0 | | 3 | 0.0 | 3 | 0.1 |
| Disorders of sexual preference | F65 | 0 | 0.0 | 0 | 0.0 | | 1 | 0.0 | 1 | 0.0 |
| Psychological and behavioural disorders associated with sexual development and orientation | F66 | 2 | 0.1 | 3 | 0.2 | | 66 | 0.8 | 0 | 0.0 |
| Other disorders of adult personality and behaviour | F68 | 8 | 0.3 | 0 | 0.0 | | 17 | 0.2 | 2 | 0.1 |
| Unspecified disorder of adult personality and behaviour | F69 | 0 | 0.0 | 0 | 0.0 | | 11 | 0.1 | 7 | 0.2 |
| Hyperkinetic disorders | F90 | 16 | 0.5 | 6 | 0.5 | | 1294 | 16.2 | 503 | 14.9 |
| Unspecified mental disorder | F99 | 259 | 8.7 | 104 | 8.1 | | 853 | 10.7 | 382 | 11.3 |

SUD = substance use disorder

*Any mental disorder (excl. SUDs)
